# Differential combination immunotherapy requirements for inflamed (warm) tumors versus T cell excluded (cool) tumors: engage, expand, enable, and evolve

**DOI:** 10.1136/jitc-2020-001691

**Published:** 2021-02-18

**Authors:** Kellsye P Fabian, Michelle R Padget, Rika Fujii, Jeffrey Schlom, James W Hodge

**Affiliations:** Laboratory of Tumor Immunology and Biology, Center for Cancer Research, National Cancer Institute, Bethesda, Maryland, USA

**Keywords:** immunotherapy, immunomodulation, vaccination, translational medical research, cytokines

## Abstract

**Background:**

Different types of tumors have varying susceptibility to immunotherapy and hence require different treatment strategies; these cover a spectrum ranging from ‘hot’ tumors or those with high mutational burden and immune infiltrates that are more amenable to targeting to ‘cold’ tumors that are more difficult to treat due to the fewer targetable mutations and checkpoint markers. We hypothesized that an effective anti-tumor response requires multiple agents that would (1) *engage* the immune response and generate tumor-specific effector cells; (2) *expand* the number and breadth of the immune effector cells; (3) *enable* the anti-tumor activity of these immune cells in the tumor microenvironment; and (4) *evolve* the tumor response to widen immune effector repertoire.

**Methods:**

A hexatherapy combination was designed and administered to MC38-CEA (warm) and 4T1 (cool) murine tumor models. The hexatherapy regimen was composed of adenovirus-based vaccine and IL-15 (interleukin-15) superagonist (N-803) to engage the immune response; anti-OX40 and anti-4-1BB to expand effector cells; anti-PD-L1 (anti-programmed death-ligand 1) to enable anti-tumor activity; and docetaxel to promote antigen spread. Primary and metastatic tumor growth inhibition were measured. The generation of anti-tumor immune effector cells was analyzed using flow cytometry, ELISpot (enzyme-linked immunospot), and RNA analysis.

**Results:**

The MC38-CEA and 4T1 tumor models have differential sensitivities to the combination treatments. In the ‘warm’ MC38-CEA, combinations with two to five agents resulted in moderate therapeutic benefit while the hexatherapy regimen outperformed all these combinations. On the other hand, the hexatherapy regimen was required in order to decrease the primary and metastatic tumor burden in the ‘cool’ 4T1 model. In both models, the hexatherapy regimen promoted CD4^+^ and CD8^+^ T cell proliferation and activity. Furthermore, the hexatherapy regimen induced vaccine-specific T cells and stimulated antigen cascade. The hexatherapy regimen also limited the immunosuppressive T cell and myeloid derived suppressor cell populations, and also decreased the expression of exhaustion markers in T cells in the 4T1 model.

**Conclusion:**

The hexatherapy regimen is a strategic combination of immuno-oncology agents that can engage, expand, enable, and evolve the immune response and can provide therapeutic benefits in both MC38-CEA (warm) and 4T1 (cool) tumor models.

## Introduction

Cytotoxic T lymphocytes (CTLs) are one of the major immune effector cells against tumors and tumors can generally be stratified based on the density of T cells in the tumor bed. ‘Hot’ tumors are those with a high degree of T cell and CTL infiltration, as well as expression of interferon (IFN) signature.^1^ These T cell-inflamed tumors also often have genomic instability resulting in high mutational burden, and tumor and immune cells that express anti-programmed death-ligand 1 (PD-L1).^2^ Hot tumors are generally more responsive to immune checkpoint inhibitor (ICI) therapy.^1–3^ In contrast, ‘cold’ tumors lack infiltrating T cells and often have low tumor mutational burden.^1 2^ Without a pre-existing adaptive immune response, cold tumors are insensitive to ICI therapy and are challenging to treat. A multimodal immunotherapy combination that targets diverse immune-tumor interactions to *engage* the immune response to generate tumor-associated antigen (TAA)-specific immune effector cells, *expand* and enhance the immune effector populations, *enable* anti-tumor activity in the tumor microenvironment (TME), and *evolve* the immune repertoire may potentially be an effective approach to treat cold tumors.

T cell initiation and activity require T cell receptor engagement, costimulatory signals, and cytokines,^4^ and the combination of ICIs with immuno-oncology (IO) agents that promote these signals may improve the therapeutic benefit. Therapeutic cancer vaccines engage the anti-tumor response by activating tumor-specific T cells. The MC38-CEA model, a variant of the MC38 murine colon carcinoma model wherein the cells were engineered to express human carcinoembryonic antigen (CEA) tumor antigen and were implanted into transgenic (Tg) C57BL/6 mice that express full-length human CEA, is an ideal model to study whether immunotherapy can overcome host immune tolerance.^5^ It has been demonstrated that vaccination with CEA protein in adjuvant did not protect these animals from tumor challenge, further demonstrating immune tolerance.^6^ Notably, these tumors in this model are minimally responsive to immune checkpoint inhibition.^7^ Preclinical studies show that virus-based vaccines targeting TAAs such as human CEA in the MC38-CEA model and Twist in the 4T1 triple negative breast cancer (TNBC) model generated TAA-specific T cells.^8^ Based on this, cancer vaccines may improve ICI therapy since fully primed and committed antigen-specific T cells have been shown to be a prerequisite for programmed cell death protein-1 (PD-1) blockade to unleash anti-tumor T cell responses.^9^

Cytokines such as interleukin-15 (IL-15) have the potential to augment ICI,^7^ as well as cancer vaccine effects.^10^ N-803, which is composed of an IL-15 mutant (IL-15N72D) complexed to a dimeric sushi domain of IL-15Rα (IL-15RαSu) fusion protein, promotes CD8^+^ T cell-dependent and natural killer (NK) cell-dependent anti-tumor activity in diverse murine tumor models.^11 12^ Furthermore, N-803 in combination with PD-L1 significantly decreased tumor burden in the MC38-CEA and 4T1 tumor models compared with monotherapy with either IO agent, demonstrating the synergistic effect of these two agents in these models.^7^ In clinical trials, N-803 was well tolerated and was shown to expand NK and CD8^+^ T cell numbers, which was associated with increased serum interferon-gamma (IFN-γ) and tumor necrosis factor-alpha (TNFα).^13^

Members of the TNF receptor family, such as OX40 and 4-1BB, are costimulatory molecules that can be triggered to enhance anti-tumor activity.^14 15^ OX40 and 4-1BB expression is induced after antigen-priming and triggering their signaling pathways results in the potentiation of T cell and NK functions. Furthermore, OX40 costimulation can interfere with the function and proliferation of regulatory T cells (Tregs), thereby reducing their suppressive activity. Agonists for OX40 and 4-1BB are currently being evaluated in the clinic in combination with ICIs.^15^

Docetaxel is an anti-mitotic chemotherapeutic agent that binds to β subunits of tubulin in microtubules and prevents their depolymerization. In addition, docetaxel has also been demonstrated to increase components of antigen-processing machinery and promote calreticulin membrane translocation in the tumor cells.^16 17^ These activities were associated with T cell modulation and sensitization of tumor cells to CTL killing, thus promoting antigen cascade as a result of the release of more TAAs in the TME.

A previous study by our group demonstrated that the MC38-CEA murine colon carcinoma and the 4T1 murine TNBC models have differential response to PD-L1 blockade.^7^ PD-L1 treatment resulted in only 1/8 mice (12.5%) cured in the MC38-CEA model and 0/20 mice (0%) cured in the 4T1 model. Furthermore, in these studies the monotherapy did not suppress tumor growth or improve median overall survival of the majority of animals. These data, taken together, suggest that these tumor models reflect patients who have partial response to current approved PD1/PDL1 blockade. Combination therapy of anti-PD-L1 with other IO agents that can engage, expand, enable and evolve may improve the treatment outcome. We strategically designed a combination treatment regimen composed of adenovirus-based vaccine (Ad-CEA or Ad-Twist), N-803, OX40, 4-1BB, PD-L1, and docetaxel (also referred to as ‘hexatherapy’) and hypothesized that MC38-CEA and 4T1 tumor models will have varying response to multimodal immunotherapeutic combinations involving an ICI.

In this study, we show that MC38-CEA is a ‘warm’ tumor that is moderately sensitive to single, double, and triple modality treatments but responds best with the hexatherapy regimen. On the other hand, 4T1 is a ‘cool’ tumor that is recalcitrant to single modality treatment. Combination treatment with four to five IO agents resulted in moderate control of 4T1 primary and metastatic tumor growth. Hexatherapy outperformed all combinations tested in decreasing tumor burden in the 4T1 model. Thus, this study provides a rationale for the application of multimodal immunotherapeutic regimens composed of adenovirus-based vaccine (Ad-CEA or Ad-Twist), N-803, OX40, 4-1BB, PD-L1, and docetaxel for both warm and cool tumors for a successful anti-tumor immune response.

## Materials and methods

### Tumor cell lines and animals

4T1 was obtained from American Type Culture Collection (ATCC) and was cultured in the recommended media. Murine colon carcinoma MC38-CEA cells were cultured as described.^18^

Female Balb/c mice were obtained from the National Cancer Institute (NCI), Frederick National Laboratory for Cancer Research (Frederick, Maryland). C57BL/6 mice transgenic for human CEA (C57BL/6-CEA-Tg) were originally obtained from a breeding pair provided by Dr J Thompson (Institute of Immunobiology, University of Freiburg, Freiburg, Germany). The mice were housed and maintained under pathogen-free conditions in microisolator cages. All experimental studies were approved by and performed in accordance with the NIH Intramural Animal Use and Care Committee guidelines.

### Animal studies

MC38-CEA (3×10^5^ cells) or 4T1 (5×10^4^ cells) were subcutaneously (s.c.) injected into the flank of C57BL/6-CEA-Tg mice or the mammary fat pad of Balb/c mice, respectively, on day 0. The MC38-CEA tumor-bearing mice were vaccinated with 10^10^ viral particles (VPs) adenovirus recombinant for full length CEA (Ad-CEA), while the 4T1 tumor-bearing mice were vaccinated with 10^10^ VPs adenovirus recombinant for full length Twist (Ad-Twist) s.c. on days 7, 14, and 21 post-tumor inoculation. N-IL15 (1 µg, s.c.) was given on days 14 and 21, anti-OX40 antibody (100 µg, intraperitoneally (i.p.); Clone OX86) on days 7, 14, and 21, 4-1BB antibody (20 µg, i.p.; Clone 17B5) on days 7, 14, and 21, PD-L1 blocking antibody (200 µg, i.p.; Clone 10F.9G2; BioXcell) on days 14 and 21, and docetaxel (500 µg, i.p.; Winthrop US) on day 21. Empty adenoviral vector (10^10^ VPs, s.c.) and rat IgG1 (100 µg, i.p.; Clone TNP6A7; BioXcell), polyclonal Syrian hamster IgG (20 µg, i.p.; BioXcell), and rat IgG2b (200 µg, i.p.; Clone LTF-2; BioXcell) isotype antibodies were used as controls. The Ad-Twist, Ad-CEA and N-803 were obtained from ImmunityBio, and anti-OX86 and anti-4-1BB antibodies from Pfizer under separate Cooperative Research and Development Agreements (CRADAs). For the depletion experiment, α-CD8 antibody (100 µg, i.p.; Clone 2.43; BioXcell) was injected on days 3, 4, 5, 12, 19, and 26 post-tumor inoculation. Tumor volume was monitored and calculated using the formula: Tumor volume=length x width^2^/2. Mice were sacrificed as indicated in the figure legends or when the size of the tumor reached the ethical limit (2000 mm^3^). 4T1 pulmonary metastases were enumerated as previously described.^19^ Tumor samples were collected, mounted, and stained for multispectral imaging as previously described.^20^

### RNA analysis

Total RNA was extracted from the tumor cells or tumor explants using the RNeasy Mini kit (Qiagen) and was analyzed using the nCounter PanCancer Immune Profiling Panel (NanoString Technologies), run by the Genomics Laboratory, Frederick National Laboratory for Cancer Research. The nSolver analysis software V.4.0.70 (NanoString) was used to evaluate the data using housekeeping genes as the normalizing controls and the untreated samples as the categorical reference value.

### Flow cytometry

Single cell suspensions were prepared from spleens and primary tumors harvested on day 28. The tumors were enzymatically digested in 2 mg/mL collagenase I and 40 U/mL DNase I in RPMI and mechanically disintegrated using gentleMACS C Tubes (Miltenyi Biotec). For in vitro stimulation, 1×10^6^ splenocytes were incubated for 4–12 hours with plate-bound CD3 (1 µg/mL; 145-2 C11) and BD GolgiPlug. Blue or aqua Live/Dead Fixable Dead Cell Stain Kit (Thermo Fisher Scientific) was used to exclude dead cells from the analysis. The following antibodies were used for surface staining: CD45-AlexaFluor700 (30-F11), CD3-APC-Cy7 (145-2 C11), CD8-FITC (KT15), CD8-PE-Cy7 (53–6.7), CD8-PerCPCy5.5 7 (53–6.7), CD4-FITC (RM4-5), CD4-PECy7 (RM4-5), CD4-605 (RM4-5), CXCR3-BV650 (CXCR3-173), PD-1-BV510 (J43), CD49b-BUV395 (HMα2), CD11b-BV510 (M1/70), CD11c-APC (HL3), F4/80-BV605 (BM8), CD19-BV711, MHC Class II (I-A/I-E)-FITC (M5/114.14.2), Ly6G-BV421 (1A8), and Ly6C-PE (AL21). The FoxP3/Transcription Factor Staining Buffer set (eBioscience) was utilized to permeabilize the cells. After permeabilization, the cells were intracellularly stained with the following antibodies: Ki67-BV421 (16A8), IFNγ-PE (XMG1.2), FoxP3-PE (FJK-16s), and CTLA4-APC (UC10-4F10-11). LSRFortessa (BD Biosciences) was utilized for flow cytometry and FlowJo FACS Analysis Software (Tree Star) was employed to analyze the data. Cell populations were identified as follows: CD4^+^ T cells: live/CD45+/CD3+/CD4+/FoxP3−; CD8^+^ T cells: live/CD45+/CD3+/CD8+; Treg: live/CD45+/CD3+/CD4+/FoxP3+; NK: live/CD45/CD3−/CD49b+; granylocytic myeloid derived suppressor cells (MDSC)/neutrophils: live/CD45+/CD3−/CD11c−/CD11b+/Ly6Ghi/Ly6Clo; monocytic MDSC/monocytes: live/CD45+/CD3−/CD11c−/CD11b+/Ly6Glo/Ly6Chi; B cells: live/CD45/CD3−/CD19+; dendritic cells (DCs): live/CD45/CD3−/CD11c+/MHCII^+^; macrophages: live/CD45+/CD3−/CD11c−/CD11b+/F4/80+.

### ELISpot

Spleens were harvested and processed individually into single cell suspensions. 1×10^6^ splenocytes were plated onto wells of 96-well plates previously coated with IFN-γ capture antibody (BD Cat# 551083). C57BL/6-CEA Tg splenocytes were stimulated with one of the following H-2D^b^- or H-2K^b^-restricted peptides (10 µg/mL) for 18 hours: CEA_526-533_ (EAQNTTYL), CEA_572-579_ (GIQNSVSA), gp70 (KSPWFTTL), JAK1 (IVYLYVVCV), Ptgfr, (VITYFFGHL), HIV-gag (SQVTNPANI), while Balb/c splenocytes were stimulated with one of the following H-2L^d^-restricted peptides: Twist (LYQVLQSDEL), AH1 (SPSYVYHQF), and β-gal (TPHPARIGL). The CEA_526-533_, CEA_572-579_, gp70, Twist, β-gal, and HIV-gag peptides were synthesized by CPC Scientific and the JAK1, Ptgfr, and AH1 peptides were generated by GenScript. IFN-γ spots were detected using the BD mouse IFN-γ ELISpot (enzyme-linked immunospot) kit and developed using the BD ELISPOT AEC substrate set according to the manufacturer’s instructions. IFN-γ spots were visualized and quantified using the CTL ImmunoSpot Analyzer.

### Statistical analysis

Comparisons among groups were performed using one-way or two-way analysis of variance with Tukey’s post-hoc analysis. The arcsine transformation was applied when needed for variance stabilization. Student’s t-test was used to compare treatment groups against the control. Data were analyzed using GraphPad Prism software, V.7.0 (GraphPad Software). Differences between groups with a p value <0.05 were considered significant.

## Results

### The MC38-CEA murine colorectal carcinoma is more immune-inflamed compared with 4T1 murine triple negative breast carcinoma

**Figure 1:**
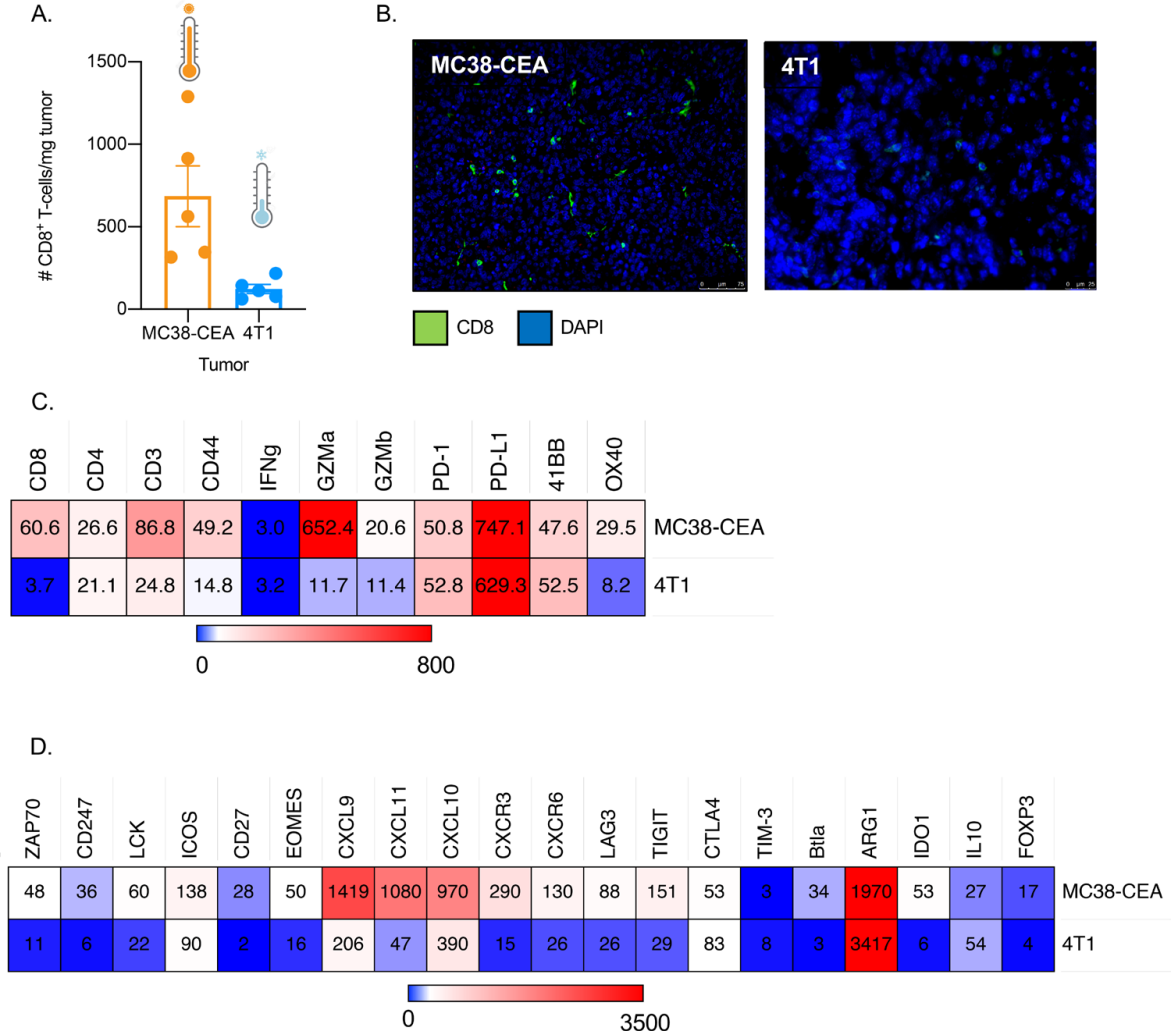
MC38-CEA colorectal carcinoma has an immune-inflamed phenotype compared with 4T1 breast carcinoma. (A, B) Female C57BL/6-CEA-Tg mice (8–12 weeks old; n=5) were implanted with 3×10^5^ MC38-CEA cells on the flank and female Balb/c mice (8–12 weeks old) were implanted with 5×10^4^ 4T1 cells on the mammary fat pad. Fourteen to fifteen days after tumor implantation, the tumors were harvested and analyzed via (A) flow cytometry and (B) immunofluorescence staining for CD8^+^ T cell infiltration. (C, D) RNA was isolated from three MC38-CEA and three 4T1 tumor explants harvested 28 days post-tumor implantation and the immune-related transcriptome for each tumor was analyzed using the nCounter PanCancer Immune Profiling Panel. Heatmap showing select genes with data presented as fold change values compared with housekeeping genes suite of that particular tumor sample on scale of 0 (light blue) to (C) 800 (red) and (D) 3500 (red). All genes reported are significantly different. CEA, carcinoembryonic antigen; Tg, transgenic.

A previous report described that PD-L1 administration did not have curative effect on 90% of MC38-CEA tumor-bearing mice and 100% of 4T1 tumor-bearing mice that were treated.^7^ Immune-inflamed tumors, also called ‘hot’ tumors, respond better to checkpoint blockade compared with ‘cold’ tumors wherein T cells are absent or excluded.^1 2^ To determine whether the differential response in the MC38-CEA and 4T1 tumor models may be associated with a variance in immune infiltrates, flow cytometry (figure 1A) and immunofluorescence (IF) microscopy (figure 1B) were performed. Substantially more CD8^+^ T cells were present in the MC38-CEA tumor lesion as compared with the 4T1. Furthermore, RNA analysis of the two tumor models revealed that the tumor immune microenvironment in MC38-CEA is more infiltrated and inflamed, with higher gene expression related to effector T cells, costimulatory molecules, and inflammatory cytokines and cytokines compared with 4T1 (figure 1C, D). DNA and RNA analyses also demonstrated that the parental MC38 tumor has higher tumor mutational burden than 4T1 (online supplemental table 1).^21^ These data indicate that the MC38-CEA tumor is ‘warmer’ than the 4T1 tumor. However, the MC38-CEA tumor model is not as T cell-inflamed and as sensitive to checkpoint blockade as the classically hot tumor models.^7 22^ Therefore, in this paper MC38-CEA will be referred to as a ‘warm’ tumor while 4T1 will be referred to as a ‘cool’ tumor.

10.1136/jitc-2020-001691.supp1 Supplementary data

### The hexatherapy treatment regimen results in enhanced therapeutic effects in the ‘warm’ MC38-CEA model

**Figure 2:**
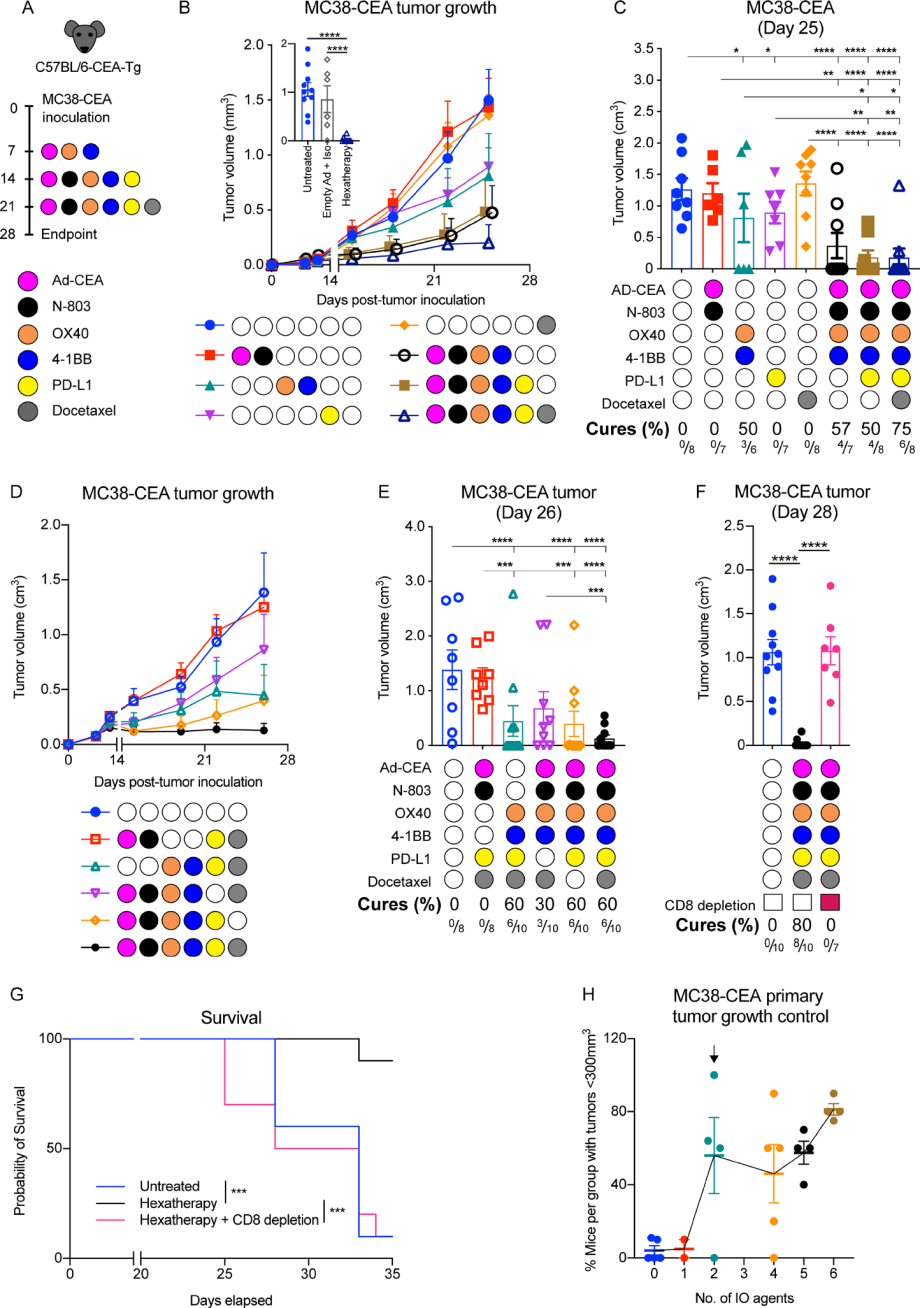
The hexatherapy treatment regimen results in enhanced therapeutic effects in the ‘warm’ MC38-CEA model. (A) Female C57BL/6-CEA-Tg mice (8–12 weeks old) were inoculated s.c. on the flank with 3×10^5^ MC38-CEA cells on day 0. Ad-CEA (1×10^10^ VP) was administered s.c. on days 7, 14, and 21; 1 µg N-803 s.c. on days 14 and 21; 100 µg anti-OX40 and 20 µg anti-4-1BB i.p. on days 7, 14, and 21; 200 µg anti-PD-L1 i.p. on days 14 and 21; and 500 µg docetaxel on day 21. In this hexatherapy regimen, the IO agents were grouped into four modalities: Ad-CEA+N-803, OX40+4-1BB, PD-L1, and docetaxel. (B, C) The single modality treatments were cumulatively combined and used to treat MC38-CEA tumor-bearing mice (n=6–8/group) using the schedule described above. Tumor volumes were monitored; (B) tumor growth curve and (C) mean tumor volumes on day 25 were plotted. Another set of MC38-CEA tumor-bearing mice (n=6–10/group) were treated with the hexatherapy regimen or with corresponding empty adenoviral vector and antibody isotype. Tumor volumes on day 28 were plotted (B inset). (D, E) MC38-CEA tumor-bearing female C57BL/6-CEA-Tg mice (n=8–10/group) were treated with the hexatherapy regimen or the hexatherapy regimen minus one treatment modality. Tumor volumes were monitored; (D) tumor growth curve and (E) mean tumor volumes on day 26 were plotted. (F, G) The hexatherapy regimen was administered to CD8^+^ T cell-depleted MC38-CEA tumor-bearing female C57BL/6 mice (n=6–10). (F) Tumor volumes on day 28 and (G) survival were monitored. The same untreated and hexatherapy-treated groups are presented in figure 2B inset and figure 2F. (H) For each treatment combination tested in the MC38-CEA model, the percentage of mice with tumor volume <300 mm^3^ was calculated and plotted against the number of IO agents received. Meta-analysis of three to four independent experiments is shown. Statistical test: Analysis of variance with Tukey’s post hoc test. Error bars, SEM. *p<0.05; **p<0.01; ***p<0.001; ****p<0.0001. CEA, carcinoembryonic antigen; IO, immuno-oncology; i.p., intraperitoneal; s.c., subcutaneous; Tg, transgenic; VP, viral particle.

We hypothesize that MC38-CEA and 4T1 tumor models will have varying responses to multimodal immunotherapeutic combinations involving an ICI. To test this hypothesis, MC38-CEA tumor-bearing CEA-Tg C57BL/6 mice were treated with Ad-CEA vaccine, N-803, OX40, 4-1BB, PD-L1, and docetaxel (figure 2A). The anti-tumor agents were grouped according to their primary function and each group was considered as a single treatment modality. The groups were: (1) Ad-CEA+N-803 to stimulate antigen-specific T cells (engage), (2) anti-OX40+anti-4-1BB to augment the activity of activated T cells (expand), (3) PD-L1 blockade to inhibit inhibitory signals (enable), and (4) docetaxel to cause immunogenic cell death that results in the release of more TAAs (evolve).

Treatment sequence and timing are critical factors in the success of combination immunotherapy. In this study, a ‘minimal patient visit’ schedule was adopted wherein the animals were treated to pattern what occurs in the clinic where patients are given immuno-oncology agents on certain days (typically at 1 or 2 week intervals) each cycle. In our previous study (accepted *manuscript; published online DOI:10.1158/2326-6066.CIR-20-0638),* we determined that the effect of Ad-CEA+N-803 was maximized when N-803 was applied after the primary immunization. Our group has also previously shown that optimal enhancement of immune response occurs when docetaxel is given after vaccination and that when docetaxel is administered prior to vaccination, the chemotherapy inhibits viral infection or transgene expression of the recombinant vaccine.^16^ Others have shown that concurrent injection of OX40 agonist and PD-1 blocking antibody attenuates the OX40-induced anti-tumor effect but delayed and sequential PD-1 administration synergizes with OX40, resulting in significant increases in therapeutic efficacy.^23 24^ Hence, for this study, even though PD-L1 was used, the ICI treatment commenced a week after the first OX40 injection.

The animal cohorts were given either single modality treatments or combination therapies in a cumulative fashion, namely Ad-CEA+N-803 and OX40+4-1BB (engage and enhance), Ad-CEA+N-803, OX40+4-1BB, and PD-L1 (engage, enhance, enable), or Ad-CEA+N-803, OX40+4-1BB, PD-L1, and docetaxel (engage, enhance, enable, evolve; hereinafter referred to as ‘hexatherapy’; online supplemental figure 1). Single modality treatment with PD-L1 blockade (p=0.0134) and OX40+4-1BB agonists (p=0.0046) resulted in delayed MC38-CEA tumor growth, while Ad-CEA+N-803 and docetaxel (p>0.05) treatments had little to no effect (figure 2B, C). The Ad-CEA+N-803+OX40+4-1BB combination also inhibited tumor growth compared with control (p<0.0001); however, it was not different to that observed with OX40+4-1BB treatment (p=0.6625), indicating that most of the therapeutic activity may be due to the costimulatory molecule agonists in this combination. On the other hand, the Ad-CEA+N-803+OX40+4-1BB+PD-L1 combination resulted in significant tumor growth inhibition compared with control (p<0.001) and OX40+4-1BB (p=0.0416) and PD-L1 (p=0.0063) single modality treatments, but the frequency of cured animals remained at 50% (3/6 mice). Treatment with the hexatherapy regimen, however, was able to significantly suppress tumor growth compared with control (p<0.0001) and compared with all single modality treatments, including PD-L1 blockade (p=0.0033) and OX40+4-1BB agonists (p=0.0268). Furthermore, the hexatherapy regimen resulted in the highest frequency (6/8 mice, 75%) of cured established tumors compared with any of the treatments. Treatment with a combination of appropriate control adenovirus vector and isotype antibodies had no therapeutic effect (figure 2B inset; online supplemental figure 2). The tumor growth data suggest that even though OX40+4-1BB or PD-L1 confers some degree of anti-tumor protection, the additional agents in the hexatherapy regimen provided a substantial contribution to the enhanced therapeutic benefit in the MC38-CEA tumor-bearing mice.

Next, we compared the efficacy of the hexatherapy regimen versus hexatherapy regimen minus one treatment modality (ie, the hexatherapy combination without OX40+4-1BB, Ad-CEA+N-803, PD-L1, or docetaxel) to assess the contribution of each treatment modality to the anti-tumor effect of the hexatherapy combination (figure 2D, E). Exclusion of OX40+4-1BB and PD-L1 treatment modalities from the hexatherapy regimen abrogated the anti-tumor therapeutic benefit. Interestingly, treatment with the hexatherapy regimen minus PD-L1 blockade did not statistically suppress tumor growth but still led to 30% tumor regression. This suggests that OX40+4-1BB and PD-L1 are the main drivers of tumor growth control in the hexatherapy regimen.

Next, we investigated the role of CD8^+^ T cells in the efficacy of the hexatherapy regimen. The treated animals were given CD8-depleting antibodies, which was confirmed via flow cytometry to deplete the CD8^+^ by 95%–100% in the blood. The depletion study showed that in the absence of CD8^+^ T cells, the hexatherapy regimen failed to control tumor growth (p*<*0.0001) and prevented the animals from rejecting the MC38-CEA tumors (figure 2F; online supplemental figure 2). In addition, on CD8^+^ T cell depletion, the median overall survival was significantly decreased (figure 2G), indicating the therapeutic benefit of the hexatherapy regimen is dependent on the CD8^+^ T cell activity.

The data suggest that for the ‘warm’ MC38-CEA tumor model, the full hexatherapy regimen may not be necessary for tumor growth inhibition. When a meta-analysis of all the repeated hexatherapy studies for MC38-CEA was performed, it revealed that a combination of two components of the hexatherapy regimen may be sufficient to allow a tumor growth control (tumors that are smaller than 300 mm^3^) in a high percentage of mice (56%) (figure 2H).

### Treatment with the hexatherapy regimen induces robust anti-tumor CD4^+^ and CD8^+^ T cell responses in the ‘warm’ MC38-CEA tumor model

**Figure 3:**
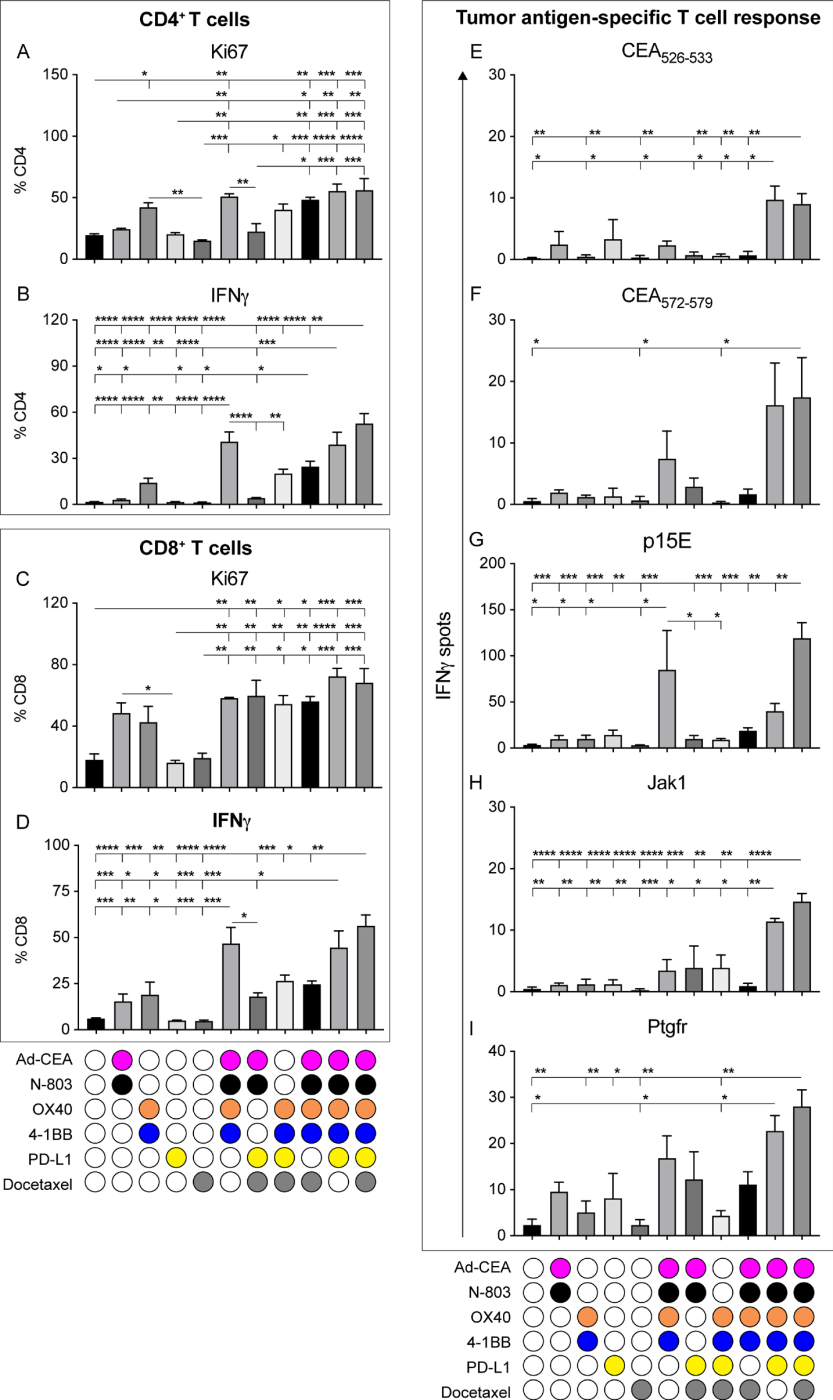
Treatment with hexatherapy induces enhanced CD4^+^ and CD8^+^ T cell activity in the ‘warm’ MC38-CEA tumor model. In a separate experiment, MC38-CEA tumor-bearing mice (n=6–10/group) were treated as in figure 2A. Three spleens from each animal cohort were collected on day 28 (7 days after the last treatment). (A–D) Splenocytes from different treatment groups were stimulated in vitro with 1 µg/mL plate-bound CD3 antibody for 4 hours. Intracellular expression of Ki67 and IFNγ in (A, B) CD4^+^ T cells and (C, D) CD8^+^ T cells was analyzed by flow cytometry as frequency of CD4^+^ and CD8^+^ T cells, respectively. (E–I) 1×10^6^ splenocytes were stimulated with H2-K^b^-restricted peptide epitopes for (E, F) CEA, (G) gp70 (p15E), the neo-epitopes (H) JAK1 and (I) Ptgfr, and HIV-gag. Antigen-specific IFN-γ production was measured via ELISpot. HIV-gag values were subtracted from the values obtained with the other antigens to normalize the data. Statistical test: Analysis of variance with Tukey’s post hoc test. Error bars, SEM. *p<0.05; **, p<0.01; ***p<0.001; ****p<0.0001. CEA, carcinoembryonic antigen; IFN, interferon; PD-L1, programmed death-ligand 1.

To assess T cell activation in the periphery, a set of MC38-CEA tumor-bearing mice were treated as described in figure 2A and spleens were harvested from treated mice at day 28, stimulated in vitro with CD3 antibody, and evaluated for CD4^+^ and CD8^+^ T cell responses. Compared with control, treatment with OX40+4-1BB, as a doublet (p=0.0410) or in combination with Ad-CEA+N-803 (p=0.0016), significantly increased the proliferative capacity (ie, Ki67 expression) of CD4^+^ T cells (figure 3A). Likewise, the hexatherapy regimen increased CD4^+^ T cell proliferation and removal of either the OX40+4-1BB or Ad-CEA+N-803 components from the regimen brought the proliferation back down to the same magnitude as the control. Similarly, treatment combinations that contained OX40+4-1BB and Ad-CEA+N-803 modalities increased IFN-γ production by CD4^+^ T cells (figure 3B). Both of these modalities are important in CD4^+^ T cell IFN-γ production as the exclusion of OX40+4-1BB or Ad-CEA+N-803 decreased the cytokine levels. Tumor growth control was still observed in the hexatherapy minus OX40+4-1BB and hexatherapy minus Ad-CEA+N-803 groups (figure 2B–E), even though peripheral CD4^+^ T cell activity was not profound in these cohorts.

CD8^+^ T splenocytes from animal cohorts that received single modality treatments, including OX40+4-1BB and PD-L1 treatment, which resulted in MC38-CEA growth suppression (figure 2B, C), did not express significantly increased Ki67 or IFN-γ compared with control (figure 3C, D). Conversely, the hexatherapy regimen and other combinations involving two or more treatment modalities promoted Ki67 expression in CD8^+^ T cells (figure 3C). Treatment with hexatherapy and multimodal combinations without docetaxel promoted IFN-γ production in CD8^+^ T cells (figure 3D). These CD4 and CD8 data indicate that in the periphery, engaging and enhancing the immune response adenovirus-based vaccine, N-803, and OX40 and 4-1BB agonists at the minimum results in the activation of effector T cells in the MC38-CEA model.

To evaluate antigen-specific responses, splenocytes from the different groups were stimulated in vitro with MHC class I H-2^b^ restricted peptide epitopes for the tumor antigens CEA_526-533_ and CEA_572-579_, the endogenous retroviral tumor antigen gp70 (p15E), and MC38-associated neoepitopes for Jak1 and Ptgfr.^21^ IFN-γ ELISpot showed that the hexatherapy regimen promoted development of CD8^+^ T cell populations targeting all the immunogenic epitopes tested (figure 3E–I). Treatment with the combination of Ad-CEA+N-803, OX40+4-1BB, and PD-L1 induced the activation of CEA_526-533_-specific, Jak1-specific and Ptgfr-specific responses (figure 3E, H, I). The combination of Ad-CEA+N-803 and OX40+4-1BB allowed for the stimulation of p15E-specific immunity (figure 3G). These findings imply that vaccine-directed anti-CEA response and antigen cascade are more robust with hexatherapy treatment compared with any of the combinations performed. However, due to the small sample size, it cannot be established whether the tumor rejection observed in figure 2 is correlated to the number of tumor-specific T cells that were activated by the hexatherapy regimen in the periphery in the individual animals.

### The hexatherapy regimen results in enhanced therapeutic effects in the ‘cool’ 4T1 tumor model

**Figure 4:**
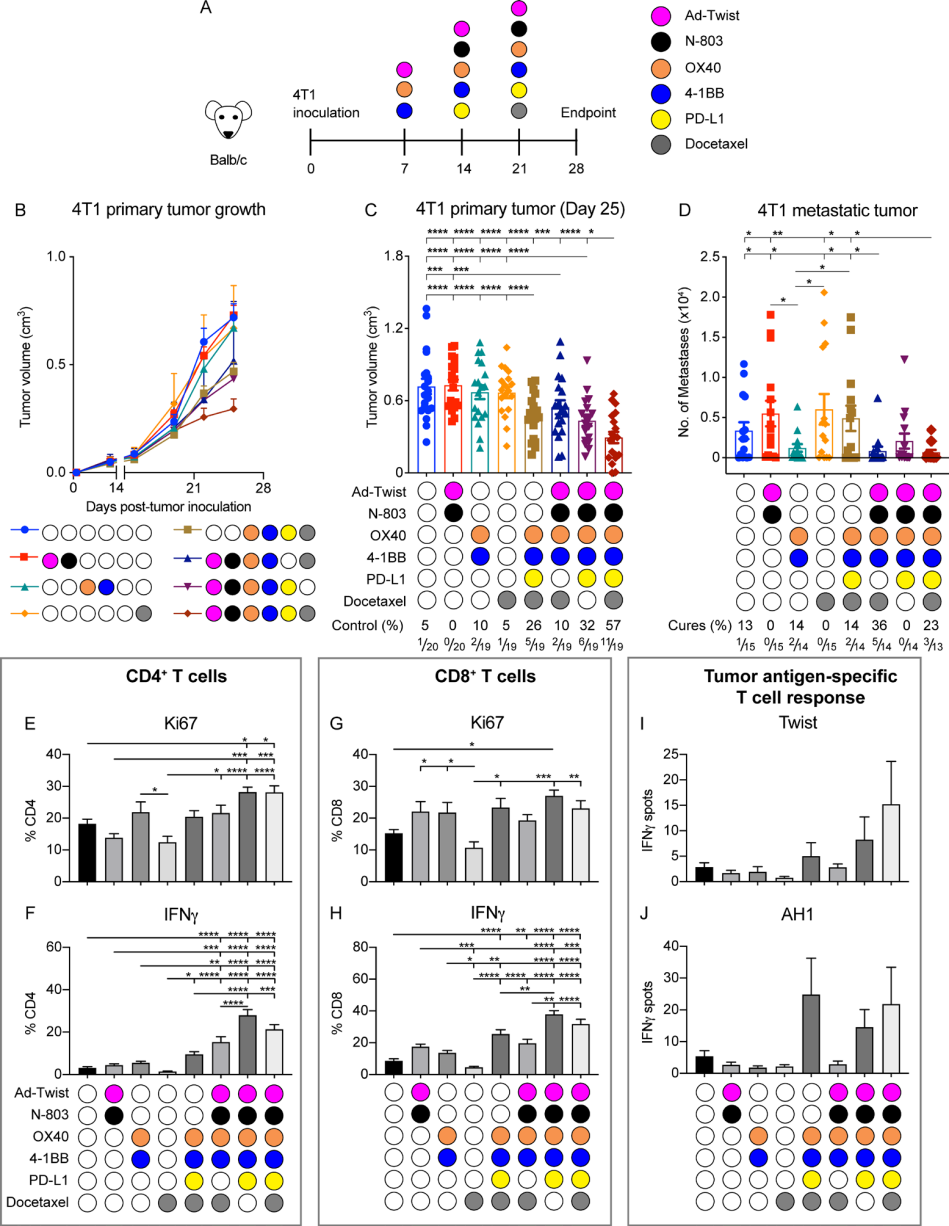
The hexatherapy regimen results in enhanced therapeutic effects associated with expanded effector immune cell populations in the ‘cool’ 4T1 tumor model. (A) Female Balb/c mice (8–12 weeks old) were implanted with 5×10^4^ 4T1 cells on the mammary fat pad on day 0. Ad-Twist (1×10^10^ VP) was administered s.c. on days 7, 14, and 21; 1 μg N-803 s.c. on days 14 and 21; 100 µg anti-OX40 and 20 µg anti-4-1BB i.p. on days 7, 14, and 21; 200 µg anti-PD-L1 i.p. on days 14 and 21; and 500 µg docetaxel on day 21. In this hexatherapy regimen, the IO agents were grouped into four modalities: Ad-CEA+N-803, OX40+4-1BB, PD-L1, and docetaxel. (B–D) 4T1 tumor-bearing mice were treated with the hexatherapy regimen or hexatherapy regimen minus one treatment modality (n=18–20/group). (B) Primary tumor volumes were monitored and (C) mean tumor volumes on day 25 were plotted. On day 28 post-tumor implantation the lungs (n=13–15/group) were collected from the different animal treatment cohorts. (D) Single cell suspension samples of lungs were incubated in complete RPMI supplemented with 6 µM 6-thioguanine for 10–12 days, after which clonogenic metastatic cell colonies were enumerated. Another set of 4T1 tumor-bearing female Balb/c mice (n=8–10/group) were treated as described above. (E–H). On day 28, splenocytes from the different treatment groups were harvested and stimulated in vitro with 1 µg/mL plate-bound CD3 antibody overnight. Intracellular expression of Ki67 and IFN-γ in (E, F) CD4^+^ T cells and (G, H) CD8^+^ T cells was analyzed by flow cytometry as frequency of CD4^+^ and CD8^+^ T cells, respectively. (I, J) The splenocytes were also stimulated in vitro with H2-K^d^-restricted peptide epitopes for (I) Twist, (J) AH1 and β-gal. Antigen-specific IFN-γ production was measured via ELISpot. β-gal values were subtracted from the values obtained with the other antigens to normalize the data. Statistical tests: Analysis of variance with Tukey’s post hoc test for group analyses. Student’s t-test for comparing two groups. Error bars, SEM. *p<0.05; **p<0.01; ***p<0.001; ****p<0.0001. CEA, carcinoembryonic antigen; IFN, interferon; IO, immuno-oncology; PD-L1, programmed death-ligand 1; s.c., subcutaneous; VP, viral particle.

Next, the hexatherapy regimen was applied to the TNBC murine tumor model, 4T1 (figure 4A), which is less immune-infiltrated compared with MC38-CEA (figure 1). Twist, a transcription factor implicated in epithelial–mesenchymal transition (EMT), is moderately expressed in primary 4T1 tumors and highly expressed in 4T1 lung metastases, and it has been previously shown that Twist is a viable vaccine target.^25^ Hence, we utilized adenovirus recombinant for Twist (Ad-Twist) for this tumor model. The 4T1-bearing mice were given single modality treatment, hexatherapy or hexatherapy minus one treatment modality. Due to the reported toxicity of the PD-L1 blocking antibody (clone 10F.9G2) in the 4T1 model,^26^ anti-PD-L1 monotherapy was excluded from the experiment. Unlike in the MC38-CEA tumor model, none of single modality treatments resulted in 4T1 primary tumor growth regression (figure 4B, C). However, treatment with the hexatherapy regimen minus one treatment modality combinations resulted in the inhibition of primary tumor growth compared with control and single modality treatments. On the other hand, the hexatherapy regimen outperformed all the groups in controlling 4T1 primary tumor growth, suggesting that superior anti-tumor benefit was achieved in the ‘cool’ 4T1 model when the immune response was engaged, enhanced, enabled, and evolved with a multifaceted immunotherapeutic combination.

Next, the effect of the multimodal therapies on metastatic formation in the lungs in the 4T1 tumor model was determined. Only the hexatherapy regimen and combinatorial therapy composed of Ad-Twist+N-803, OX40+4-1BB, and docetaxel decreased metastatic lung colonies (figure 4D). Treatment with hexatherapy without Ad-Twist+N-803 and without docetaxel did not hinder metastatic tumor formation even though these combinations resulted in 4T1 primary tumor suppression.

Next, we examined the effects of the hexatherapy combination on peripheral T cell activity. Splenocytes were harvested from the treated animals and were stimulated with CD3 antibody. Compared with control, treatment with hexatherapy (p=0.030) and hexatherapy without docetaxel (p=0.029) increased Ki67 expression in CD4^+^ T cells (figure 4E). These treatments, together with hexatherapy minus PD-L1 (p<0.0001 for all groups), also resulted in the significant production of IFN-γ in the CD4^+^ T cells (figure 4F). On the other hand, none of the administered treatments had an impact on the proliferative capacity of the CD8^+^ T cells (figure 4G). All of the multimodal combinations (p<0.01 for all groups) promoted CD8^+^ T cell production of IFN-γ (figure 4H). To investigate the antigen-specific CD8^+^ T cell response, the splenocytes were also stimulated with MHC class I H2^d^-restricted peptide epitopes for the vaccine target Twist and the endogenous retroviral tumor antigen gp70 (AH1). IFN-γ ELISpot did not show statistical increase in antigen-specific T cells; however, it showed that the number of Twist-specific CD8^+^ T cells quintupled on hexatherapy treatment while it almost tripled with the hexatherapy minus docetaxel treatment (figure 4I). Meanwhile, CD8^+^ T cell response against AH1 quadrupled with hexatherapy and hexatherapy minus Ad-Twist+N-803 treatments (figure 4J). Overall, the in vitro stimulation studies demonstrate that multimodal immunotherapies tested can induce peripheral CD4^+^ and CD8^+^ T cell activity in the 4T1 tumor model. Notably, the hexatherapy regimen can increase T cell responses directed against the vaccine-targeted and cascade antigens.

The effect of the different treatment combinations on the tumor immune landscape was examined next. Single modality treatment did not modify the distribution of the different immune populations in the spleen (online supplemental figure 3A) or tumor (online supplemental figure 3B). However, the multimodal treatments, including the hexatherapy regimen, resulted in an altered immune composition especially in the tumor (online supplemental figure 3B). The multimodal treatments favored the expansion of effector immune cell populations, specifically that of CD4^+^ and CD8^+^ T cells. Even though NK cells contribute to anti-tumor immunity and can be activated with N-803, OX40, and 4-1BB,^12 27^ we did not observe a change in frequency in this population across the treatment groups. On the other hand, the MDSC populations contracted on the hexatherapy treatment. The interrogation of tumor immune infiltrates indicated that the hexatherapy regimen modifies the immune landscape to favoring the effector populations over immunosuppressive cell types.

### Treatment with hexatherapy results in tumor-infiltrating T cells that are more proliferative and less exhausted in the ‘cool’ 4T1 tumor model

**Figure 5:**
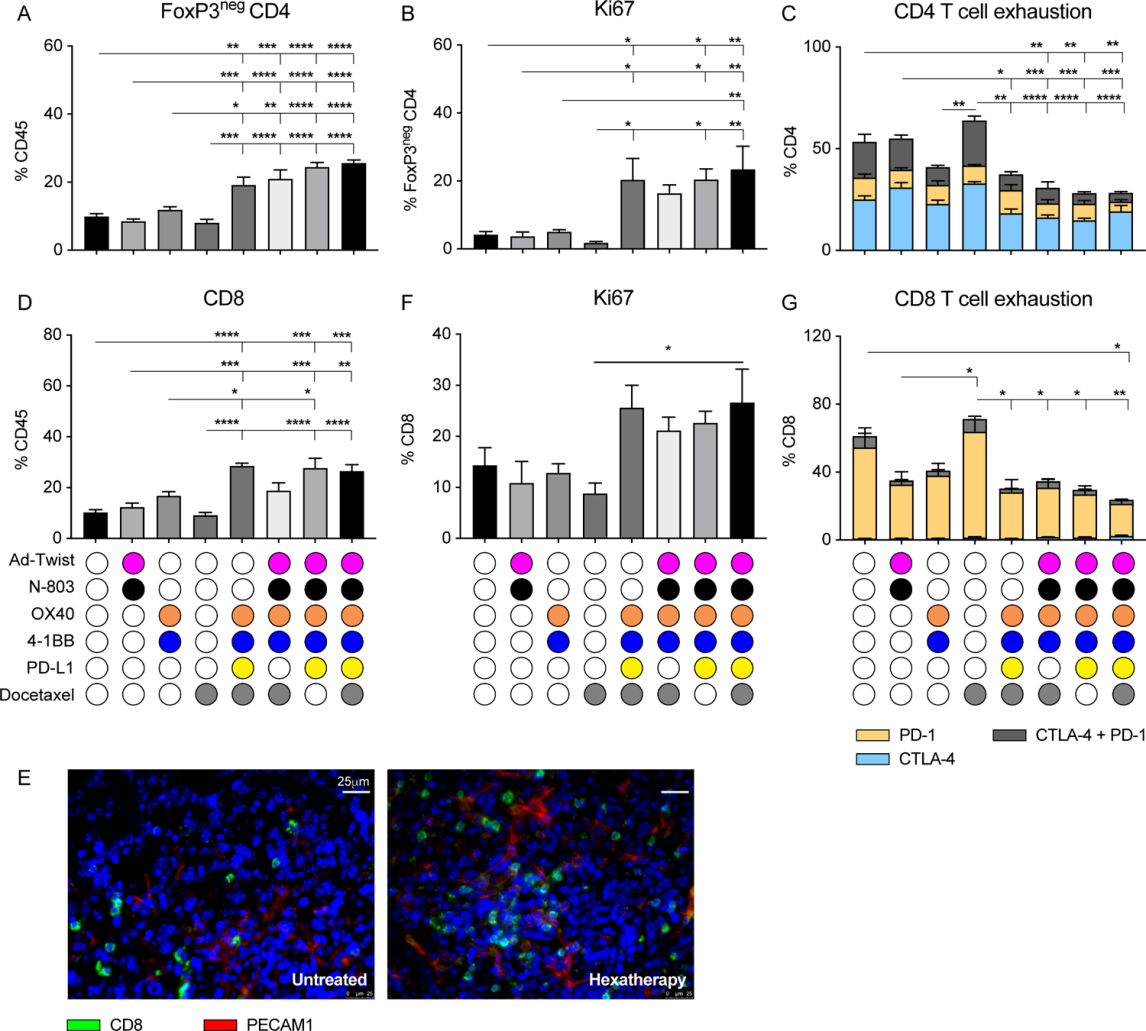
Treatment with the hexatherapy regimen results in tumor-infiltrating T cells that are more proliferative and less exhausted in the ‘cool’ 4T1 tumor model. Primary tumors (n=4–5/group) from figure 4B, C were collected on day 28 post-tumor implantation (7 days after the last treatment). (A–C) Flow cytometry was performed to determine (A) the frequency of FoxP3^neg^ CD3^+^CD4^+^ T cells in the CD45^+^ tumor-infiltrating immune population, (B) the frequency of Ki67^+^ expression in the FoxP3^neg^ CD4^+^ T cells, and the (C) frequency of CTLA-4 and PD-1 expression in the CD4^+^ T cells. (D) Likewise, flow cytometry was performed to determine the frequency of CD3^+^CD8^+^ T cells in the CD45^+^ compartment. (E) Immunofluorescence staining of CD8^+^ T cells (green) and PECAM1^+^ cells (red) was performed on untreated and hexatherapy regimen-treated tumors to further elucidate T cell infiltration. (F, G) Flow cytometry was done to assess (F) the frequency of Ki67^+^ expression, and the (G) frequency of CTLA-4 and PD-1 expression in the CD8^+^ T cells. Statistical test: Analysis of variance with Tukey’s post hoc test. Error bars, SEM. *p<0.05; **p<0.01; ***p<0.001; ****p<0.0001. PD-1, programmed cell death protein-1; PECAM1^+^, platelet endothelial cell adhesion molecule-1 positive.

We next examined the ‘cool’ 4T1 tumor-infiltrating CD4^+^ and CD8^+^ T cells by performing flow cytometry on bulk tumors. There, all the different multimodal combination treatments tested promoted the increase in the frequency of effector CD4^+^ T cells in the tumor lesion (figure 5A). These are the same groups in which 4T1 primary tumor growth regression was observed (figure 4B, C). More importantly, the infiltrating CD4^+^ T cells have improved proliferative potential (figure 5B) while at the same time have reduced expression of the T cell exhaustion markers PD-1 and CTLA-4^28^ with the different multimodal combination therapy (figure 5C).

The tumor-infiltrating CD8^+^ T cells were generally amplified with multimodal therapy, notably with the hexatherapy regimen, hexatherapy minus docetaxel, and hexatherapy minus Ad-Twist+N-803 treatments (figure 5D). CD8^+^ T cell infiltration in the hexatherapy group was confirmed via IF microscopy (figure 5E). The IF images show that CD8^+^ T cells were non-adjacent to the platelet endothelial cell adhesion molecule-1 positive (PECAM1^+^) vascular compartment and were situated in the extravascular space. Although not statistically significant, the Ki67^+^CD8^+^ T cell population doubled in the hexatherapy-treated cohort compared with control (figure 5F). Hexatherapy treatment also resulted in diminished CD8^+^ T cell exhaustion as represented by the decrease in frequency of CTLA-4^+^, PD-1^+^ and CTLA-4^+^PD-1^+^ CD8^+^ T cells (figure 5G). Hexatherapy treatment improved tumor growth control in primary and metastatic 4T1 settings compared with all the combinations tested (figure 4B–D) by promoting effector CD4^+^ and CD8^+^ cells while at the same time limiting T cell exhaustion.

In spite of immune activation, the hexatherapy regimen was generally well-tolerated by the animal cohorts, with no negative on-study observations or weight loss. Organ pathology also showed that the kidney, heart, duodenum, and brain exhibited normal cellularity and architecture with the hexatherapy regimen (online supplemental figure 4 and online supplemental table 2). All the treatments, including hexatherapy, were associated with some degree of liver inflammation.

### Multimodal immunotherapy resulted in decreased immunosuppressive cell populations in the 4T1 tumor model

**Figure 6:**
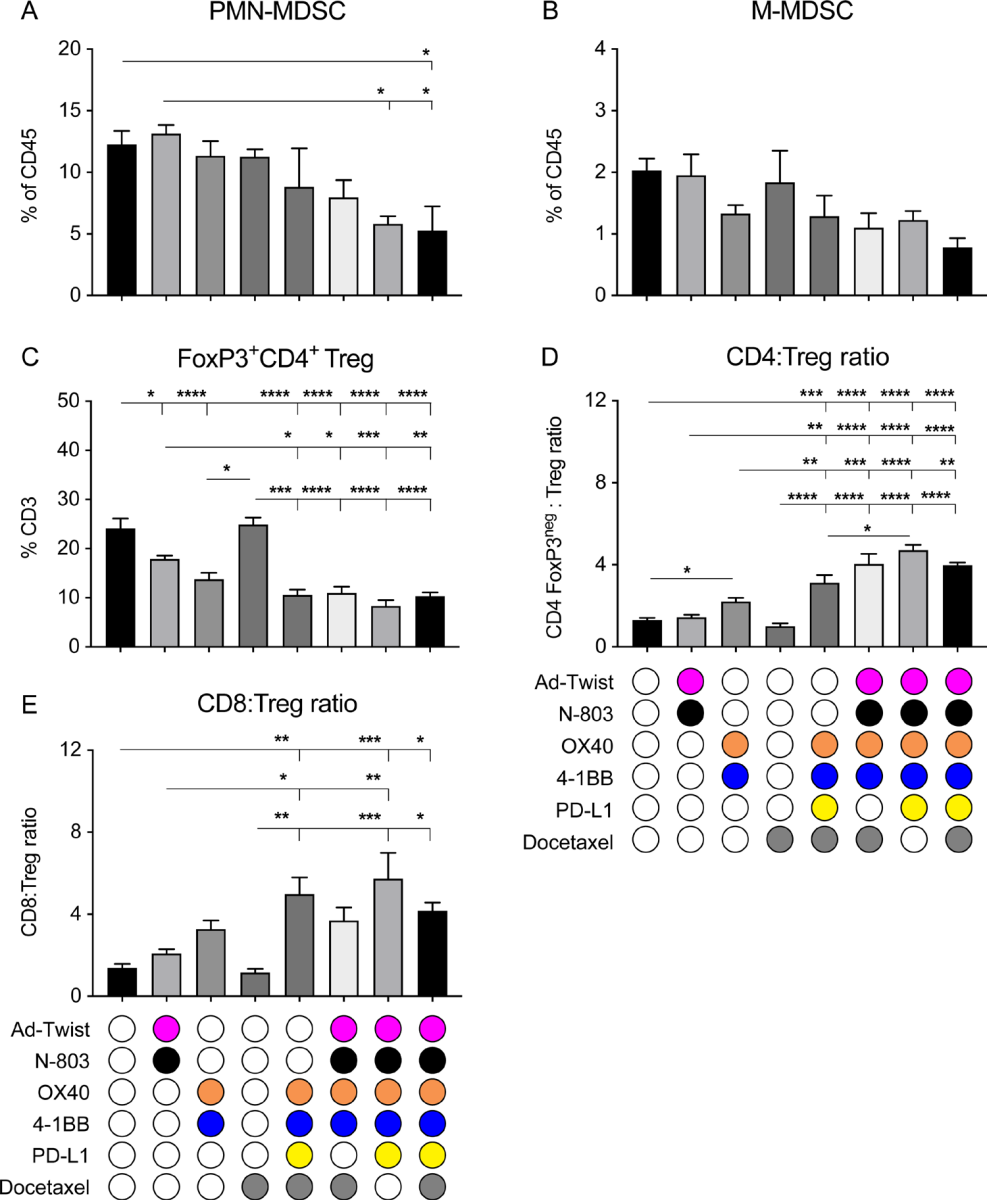
The hexatherapy treatment regimen reduces PMN-MDSC and improves effector T cell-to-Treg cell ratios in the 4T1 tumor microenvironment. (A–C) Primary tumors (n=4–5/group) from figure 4B, C were collected on day 28 post-tumor implantation (7 days after the last treatment) and were assessed through flow cytometry for frequency of (A) PMN-MDSC (CD11b^+^Ly6G^+^Ly6C^lo^), (B) M-MDSC (CD11b^+^Ly6G^−^Ly6C^high^), and (C) Tregs (FoxP3^+^CD4^+^ T cells) in the CD45^+^ population. (D, E) Flow cytometric analysis was used to determine the (D) CD4-to-Treg ratio and (E) CD8-to-Treg ratio in the tumor. Analysis of variance with Tukey’s post hoc test. Error bars, SEM. *p<0.05; **p<0.01; ***p<0.001; ****p<0.0001. M-MDSC, mononuclear myeloid derived suppressor cell; PD-L1, programmed death-ligand 1; PMN-MDSC, polymorphonuclear MDSC; Treg, regulatory T cell.

We next studied the immunosuppressive cell populations in the tumor lesions of the treated animals. As noted in online supplemental figure 3B, the MDSC population contracted on hexatherapy administration. Specifically, the CD11b^+^Ly6G^high^Ly6C^low^ polymorphonuclear-MDSC (PMN-MDSC) and CD11b^+^Ly6G^-^Ly6C^high^ mononuclear MDSC (M-MDSC) populations in the 4T1 primary tumors decreased by half in the hexatherapy-treated cohort compared with control (figure 6A, B). PMN-MDSC and M-MDSC use different mechanisms of immunosuppression; therefore, limiting both populations is important to foster anti-tumor immunity.^29^ In addition, the FoxP3^+^CD4^+^ Treg population was decreased by all the multimodal combination treatments and by Ad-Twist+N-803 and OX40+4-1BB single modal therapies (figure 6C). The decrease in Tregs and the increase in CD4^+^ and CD8^+^ T cells resulted in favorable CD4-to-Treg and CD8-to-Treg ratios in the multimodal treatment groups (figure 6D, E). A high CD8-to-Treg ratio in patients with breast cancer has been correlated to high objective response rate and long progression-free survival after treatment.^30 31^

### Treatment with the hexatherapy regimen results in superior anti-tumor benefit in the ‘cool’ 4T1 tumor model

**Figure 7:**
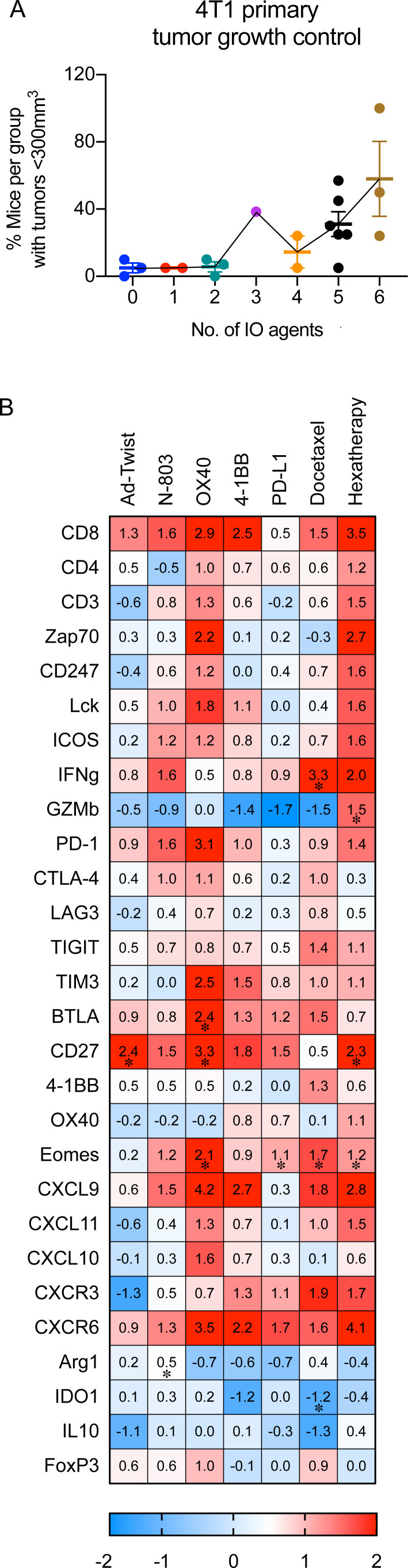
Treatment with the hexatherapy regimen results in enhanced therapeutic effects associated with expanded effector immune cell populations in the 4T1 tumor model. (A) For each treatment combination tested in the 4T1 tumor model, the percentage of mice with tumor volume <300 mm^3^ was calculated and plotted against the number of IO agents received. Meta-analysis of three independent experiments is shown. (B) 4T1 tumor-bearing female Balb/c mice (8–12 weeks old) were treated with hexatherapy regimen or single IO agent as described in figure 4A. Two to three tumor samples from each animal cohort were harvested on day 28 post-tumor implantation and were used to analyze the immune-related transcriptome using the nCounter PanCancer Immune Profiling Panel. Heatmap showing select genes with data presented as fold change on scale of −2 (blue) to +2 (red) relative to the gene expression in the untreated tumors. Statistical test: Student’s t-test. *p<0.05. IO, immuno-oncology; PD-1, programmed cell death protein-1; PD-L1, programmed death-ligand 1.

Meta-analysis of all the repeats of hexatherapy regimen studies for the ‘cool’ 4T1 tumor model shows that the hexatherapy regimen is required to achieve tumor growth control in 58% of the treated animals (figure 7A), whereas only two IO agents were needed to achieve the same rate in the MC38-CEA tumor model (figure 2H).

RNA analysis of tumors collected from hexatherapy regimen-treated animals and from monotherapy-treated animals was performed to further assess the immune response. Treatment with the Ad-Twist, N-803, OX40 agonist, 4-1BB agonist, PD-L1 inhibitor, or docetaxel as single agents had minimal to moderate observable impact on the expression of immune-related genes compared with control (figure 7B). On the other hand, hexatherapy treatment regimen correlated with an increased expression of genes associated with T cell activation, T cell effector functions, and migration to inflamed tissues/tumor, supporting the data we have described in figures 4 and 5. In addition, genes related to immunosuppression were downregulated with hexatherapy treatment, supporting our data in figure 6. The data suggest that the hexatherapy regimen altered the 4T1 tumor immune landscape, favoring an anti-tumor effector phenotype and decreasing immunosuppression.

## Discussion

ICIs are becoming one of the major cornerstones of cancer therapy. Currently, there are seven different ICIs that have been approved as first-line treatment for some indications, such as metastatic melanoma and non-small cell lung cancer, or as second-line therapy in regimen-refractory cancers.^32^ Despite its success in the clinic, the response rate to ICIs is still quite low, with a subset of patients with cancer not benefitting from the therapy. Some patients who initially respond to ICIs develop resistance and still develop progressive disease and others do not respond at all.^33^

Currently, there are no conclusive predictive biomarkers for patient response to ICIs but several factors have been linked to sensitivity to ICI. ‘Hot’ or T cell-inflamed tumors, which are those with high immune infiltrates and/or IFN signature, have been found to have higher response rates to ICIs.^1 2^ In contrast, tumors that have immune-excluded or immune-desert phenotypes are more recalcitrant to ICIs. Tumors with high tumor mutational burden (TMB) have also been correlated with objective response rate.^34^ Likewise, PD-L1 expression on tumor and immune cells has been demonstrated to be predictive of sensitivity to PD-1/PD-L1 blockade.^35^

Based on TMB and T cell-inflamed status, the MC38-CEA colorectal carcinoma model represents a ‘warm’ tumor (figure 1, online supplemental table 1)^21^ and appears to be similar to human colorectal cancers (CRC) with deficiency in mismatch repair (dMMR)/microsatellite instability-high (MSI-H). CRC with dMMR/MSI-H have high TMB and often have high immune infiltrates.^36 37^ In the clinic, monotherapy with anti-PD-1 monoclonal antibodies (mAbs), pembrolizumab and nivolumab, and combination therapy of nivolumab with the anti-CTLA-4 mAb, ipilimumab, have been approved for patients with CRC with dMMR who have progressed after treatment with first-line chemotherapy.^38^ However, only a very small subset of patients with CRC could benefit from ICI since only a small percentage of the CRC patient population has the dMMR phenotype. In contrast, our data show that the 4T1 TNBC murine model is a ‘cool’ tumor with low immune infiltrates, low immune-inflammation and low TMB (figure 1, online supplemental table 1). In the clinic, atezolizumab (anti-PD-L1) in combination with paclitaxel has been approved as treatment for patients with TNBC that expresses PD-L1.^32 39^

Many groups have reported in preclinical models and in clinical practice that combination immunotherapy is required to hit different nodes of the cancer immunity cycle. Uno *et al* described the utilization of an agonistic anti-DR5 mAb (to induce apoptosis) with two additional mAbs CD40 (for T cell activation) and CD137 (4-1BB for T cell activation).^40^ The data presented here extended that of Uno *et al*, in that we also addressed barriers to successful immunotherapy; however, we expanded on the immune areas to address to up to six IO agents which are quite distinct from that described.^40^ We utilized (1) a tumor antigen vaccine (to induce T cell responses), (2) an IL-15 superagonist (to activate T cells and NK cells), (3) an anti-4-1BB (for T cell activation), (4) an anti-OX40 (for T cell activation), (5) an anti-PD-L1 to block T cell suppression, and (6) the systemic chemotherapy docetaxel.

We hypothesized that a difference in the immune-inflammation and TMB status of MC38-CEA and 4T1 implies that the two tumor models may require different treatment combinations. For the MC38-CEA tumor, a combination of IO agents that can activate the pre-existing T cells may be sufficient for an anti-tumor response. Conversely, the 4T1 model would require a more extensive combination that would target diverse immune-tumor interactions. To achieve this, we designed a multimodal combination treatment composed of adenovirus-based TAA-targeting vaccine + N-803 to engage the effector cells, OX40+4-1BB agonists to enhance the anti-tumor activity, PD-L1 blockade to enable immune response in the TME, and docetaxel to induce immunogenic cell death and activate a different population of TAA-specific T cells (evolve). It was observed that while the 4T1 model necessitated the administration of all six agents (hexatherapy regimen) to decrease the overall tumor burden (figure 7A), significant tumor control of MC38-CEA could be achieved using a combination of two IO agents (figure 2H).

Treatment with PD-L1 and OX40+4-1BB suppressed MC38-CEA tumor growth (figure 2B, C). Both modalities are capable of enhancing and enabling the activity of the pre-existing T cells in the MC38-CEA parenchyma. Although treatments which engage the immune response like Ad-CEA+N-803 and docetaxel had minimal MC38-CEA anti-tumor effect, each agent synergized with PD-L1 and OX40+4-1BB to augment the anti-tumor outcome. Hence, MC38-CEA tumor growth control achieved with PD-L1 and OX40+4-1BB was further enhanced with the hexatherapy regimen combination (figure 2B, C). The hexatherapy regimen also improved the frequency of tumor rejection (% cures) at endpoint. However, prolonged follow-up or tumor re-challenge studies would be required to determine whether the hexatherapy treatment confers protection against tumor recurrence or outgrowth.

Cold tumors, partially represented by the 4T1 TNBC model in this study, are more challenging to treat. The lack of T cell infiltration in the cold tumor lesions may be due to one or more factors such as dearth of tumor antigens, defect in antigen processing and presentation, lack of T cell activation, and inability of T cells to home in on the tumor.^41^ The data presented indicate that single modality treatments of Ad-Twist+N-803, OX40+4-1BB, and docetaxel have no effect on 4T1 primary and metastatic growth (figure 4B–D). In contrast, treatment with the hexatherapy regimen that could address different barriers in the cancer-immunity cycle resulted in a decrease in the total tumor burden in 4T1-bearing mice. Each of the components of the hexatherapy regimen was essential in controlling primary tumor growth as the removal of one treatment modality resulted in diminished effectiveness of the combination. However, it should be noted that compared with control, the omission of one hexatherapy regimen component still resulted in tumor growth reduction. This implies that there is an overlap in the functions of the IO modalities that could compensate for the absence of some agents. Notably, treatment combinations that resulted in primary tumor growth reduction did not always translate to inhibition of metastatic formation in the lungs. For example, the combination of OX40+4-1BB, PD-L1, and docetaxel modalities resulted in decreased tumor volume but did not inhibit metastasis of 4T1 into the lungs. On the other hand, the Ad-Twist+N-803+OX40+4-1BB+docetaxel treatment only moderately decreased primary tumor volume but had a profound effect in preventing the formation of metastatic lesions. The differences in the immunological signature between primary and metastatic tumor lesion^42^ may account for the difference in response to the combinations. Ad-Twist+N-803 treatment appears to be important in the anti-metastatic activity of the hexatherapy regimen. Twist is a driver of EMT and metastatic progression and it has been shown that Twist-targeted vaccination can generate Twist-specific CD8^+^ T cells that inhibit the formation of lung metastases.^25^

The anti-tumor effect of the hexatherapy treatment is dependent on CD8^+^ T cell responses, as demonstrated by the depletion study (figure 2F, G). The hexatherapy regimen induced robust IFN-γ production in peripheral CD8^+^ T cells (figures 3D and 4F) and promoted not only vaccine antigen-specific T cells (figures 3E, F and 4I) but also T cells specific for the endogenous retroviral tumor antigen gp70 (p15E and AH1; figures 3G and 4J) and neoepitopes (Jak1 and Ptgfr; figure 3H, I). It should be noted, however, that although the hexatherapy regimen induced appreciable fold changes in the number of tumor antigen-specific T cells in the 4T1 model, they were not significant. Vaccine-directed killing of tumor cells has been demonstrated to subsequently induce antigen cascade. For example, docetaxel in combination with cancer vaccines has been shown to enhance activated CTL killing by increasing the tumor cell permeability and thus increasing tumor susceptibility to granzyme-B dependent killing, thereby potentially releasing new TAAs in the environment.^17 43^ The recalcitrance of the 4T1 model to vaccine and immunotherapy in general may explain the decreased ability of hexatherapy to promote vaccine and cascade antigen-specific T cells in the 4T1 model compared with the MC38-CEA model.

The hexatherapy regimen-driven decrease in tumor burden in the 4T1 tumor model was also associated with increased CD8^+^ T cell infiltration and proliferation, and decreased CD8^+^ T cell exhaustion in the tumor (figure 5D–G). The recruitment of effector T cells in the TME was related to the amplified expression of the chemokine receptor CXCR3 on the CD8^+^ T cells (online supplemental figure 5) and its chemokine ligands, CXCL9, CXCL10, and CXCL11^44^ in the 4T1 tumors (figure 7B). Furthermore, these CD8^+^ T cells had extravasated from the tumor blood vessel and were not localized along the border of the tumor mass (figure 5E). This outcome, and the observation that the hexatherapy regimen increased granzyme B and IFN-γ production (figure 7B), indicate that the hexatherapy regimen converted the 4T1 tumor into the infiltrated-inflamed phenotype.^45^

Immunosuppressive cells such as MDSCs and Tregs, which are associated with poor prognosis and resistance to therapy,^46^ were reduced with the hexatherapy treatment, thereby causing an improvement to the T effector-to-Treg ratio (figure 6). As with the primary tumor growth suppression, removal of one treatment modality did not nullify the immune effects seen with hexatherapy in most instances. Again, this may be due to the overlapping functions of the different treatment modalities.

Spitzer *et al*^47^ observed that the engagement of systemic immunity was a prerequisite for the initiation of the local responses critical to tumor rejection. Our data confirm and extend that of Spitzer; induction of robust induction of systemic T cell responses specific for multiple tumor antigens (figures 3E and 4I, J) was coincident with the decrease in local T cell exhaustion in the tumor microenvironment.

The effect of PD-L1 monotherapy on tumor progression was not tested on the 4T1 tumor model due to reported and observed adverse hypersensitivity reactions.^26^ Intriguingly, the combination of PD-L1 with other IO components in the hexatherapy regimen canceled PD-L1 toxicity. One of the concerns in combination therapy is the compounding immune-related adverse effects that are caused by the general, non-tumor specific activation of the immune response.^48^ Hexatherapy appeared to be well-tolerated in the 4T1-bearing Balb/c mice. Organ pathology showed normal architecture and cellularity in the kidney, heart, duodenum, and brain (online supplemental table 2 and online supplemental figure 4). All of the treatment modalities and combinations resulted in mild/moderate liver inflammation; however, this is of unknown significance as there were no associated observable signs of on-study adverse effects or weight loss. Liver inflammation has been reported as a side effect of ICI therapy and 4-1BB treatment^48^ in this study; however, it was also observed in the Ad-CEA+N-803- and docetaxel-treated groups. However, it should be noted that preclinical models do not necessarily reflect or predict clinical immune-related adverse effects (irAEs). Adverse events may not be observed in mice because mouse studies are usually short and are terminated before the onset of the irAE or because of inherent resistance of certain mouse strains to irAE induction.^49 50^

Multiple preclinical studies have shown that combination therapy targeting diverse immune-tumor interactions results in superior anti-tumor responses.^21 51^ Adaptive-design clinical trials with a sequential treatment strategy, wherein a safety signal has to be achieved first in a patient cohort before enrollment begins to another study arm that will receive an additional IO agent, will allow for the expedient and safe testing of combinatorial immunotherapy.^52^ The ongoing Quick Efficacy Seeking Trial (QuEST1; NCT03493945) that investigates the safety and efficacy of the combination of anti-tumor vaccine, TGF-β TRAP/anti-PD-L1 antibody, N-803, and epacadostat in metastatic castration-resistant prostate cancer could provide a framework for such rational adaptive-design clinical studies.

In this study, we demonstrated the differences in therapy requirements for a ‘warm’ tumor model compared with a ‘cool’ tumor. Treatment with IO agents, such as anti-PD-L1 and OX40+4-1BB agonists, that could enhance and enable pre-existing T cells in the immune-inflamed ‘warm’ MC38-CEA tumor resulted in decreased tumor burden. Treatment of MC38-CEA with the hexatherapy regimen, however, resulted in superior therapeutic benefit, indicating the importance of targeting multiple diverse immune-tumor interactions. Meanwhile, the non-inflamed ‘cool’ 4T1 tumor benefitted the most from the hexatherapy regimen that is composed of IO modalities that engage, enhance, enable, and evolve the immune response. The decrease in primary and metastatic burden was associated with increased T cell infiltration and activity, increased effector gene signature in the TME, decreased T cell exhaustion, and decreased immunosuppressive cell populations. This study provides rationale for the application of multimodal immunotherapeutic regimens for both ‘warm’ and ‘cool’ tumors for a successful anti-tumor immune response.
