# Supplementary material for: Differential combination immunotherapy requirements for inflamed (warm) tumors versus T cell excluded (cool) tumors: engage, expand, enable, and evolve
Source: J Immunother Cancer. 2021 Feb 18;9(2):e001691. doi: 10.1136/jitc-2020-001691 (PMC7896589; doi:10.1136/jitc-2020-001691)
Supplement: Supplementary data [file jitc-2020-001691supp001.pdf]

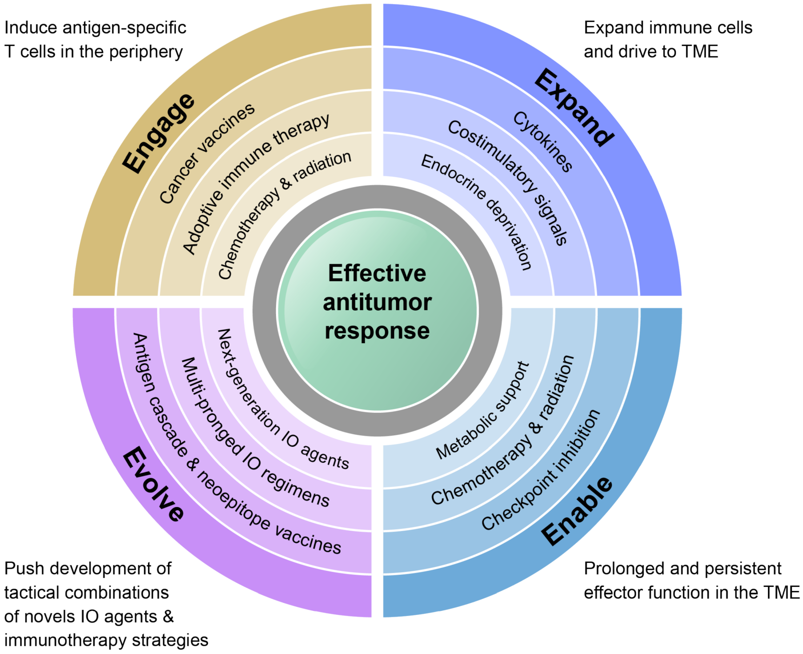

Supplemental Figure 1. Multimodal immunotherapy to *E*ngage, *E*xpand, *E*nable, and *E*volve anti-tumor immune responses. IO, immuno-oncology; TME, tumor microenvironment.

Supplemental Table 1. Characteristics of neoepitopes identified in MC38 and 4T1 tumors

	MC38	4T1 primary tumor (mammary fat pad)	4T1 metastatic tumor (lungs)
Number of samples	2	4	3
Number of nonsynonymous mutations identified by whole-genome sequencing	18114; 8946	808-1099	27-88
Number of expressed nonsynonymous mutations	7098; 3548	246-416	0-9

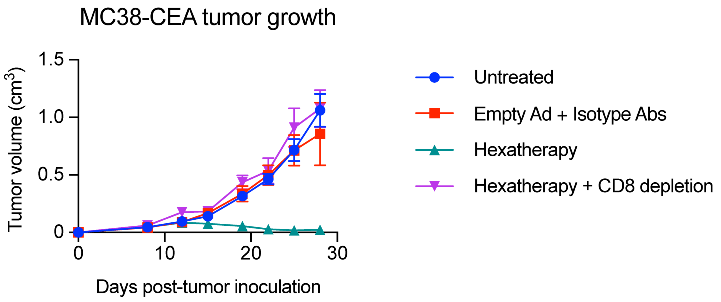

Supplemental Figure 2. MC38-CEA tumor-bearing mice were administered with empty adenovirus vector and isotype antibodies or hexatherapy (as described Figure 2A) with or without CD8 depletion antibodies. Treatment with empty adenovirus vector and isotype antibodies has no therapeutic effect. Tumor growth inhibition by the hexatherapy regimen was abrogated when $CD8^+$ T cells were depleted.

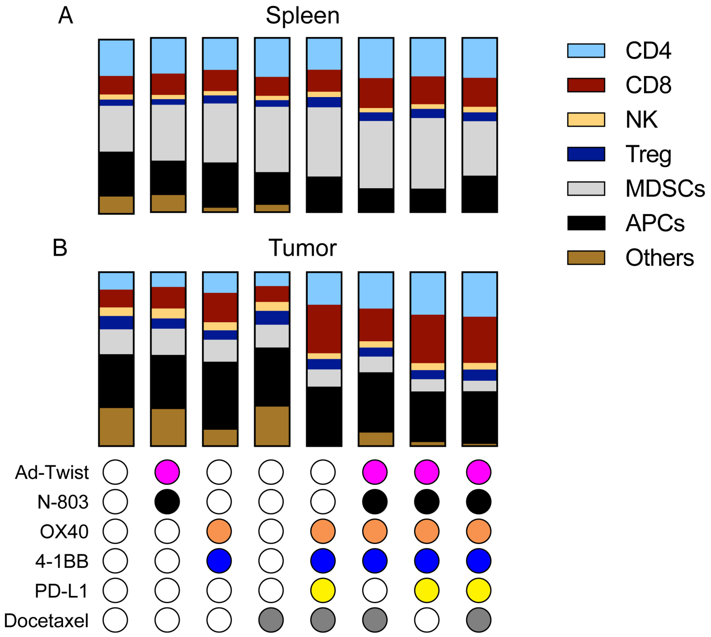

Supplemental Figure 3. (A) Spleens and (B) tumors (n=4-5/group each) were harvested from animals in Figure 4C and were analyzed for immune cell populations. The survey was performed using flow cytometry and included T cells (CD4+ and CD8+), NK cells, Tregs, MDSCs, and APCs (B cells, DCs, macrophages) as a frequency of CD45+ cells. APC, antigen-presenting cell; DC, dendritic cell i.p., intraperitoneal; NK, natural killer; s.c., subcutaneous; MDSC, myeloid derived suppressor cell; TCR, T cell receptor; Treg, regulatory T cell.

Supplemental Table 2. Tissue pathology report

Treatment	Murine Organ	Diagnosis
None	Brain	Normal (3/3)
	Duodenum	Normal (3/3)
	Heart	Normal (3/3)
	Kidney	Normal (3/3)
	Liver	Normal (3/3)
Ad-Twist + N-803	Brain	Normal (3/3)
	Duodenum	Normal (3/3)
	Heart	Normal (3/3)
	Kidney	Normal (3/3)
	Liver	Mild chronic inflammation (3/3)
OX40 + 4-1BB	Brain	Normal (3/3)
	Duodenum	Normal (3/3)
	Heart	Normal (3/3)
	Kidney	Normal (3/3)
	Liver	Moderate chronic and acute inflammation (3/3)
Docetaxel	Brain	Normal (3/3)
	Duodenum	Normal (3/3)
	Heart	Normal (3/3)
	Kidney	Normal (3/3)
	Liver	Normal (1/3); Mild inflammation (2/3)
OX40 + 4-1BB + PD-L1 + docetaxel	Brain	Normal (3/3)
	Duodenum	Normal (3/3)
	Heart	Normal (3/3)
	Kidney	Normal (3/3)
	Liver	Mild to moderate chronic and acute inflammation (3/3)
Ad-Twist + N-803 + OX40 + 4-1BB + docetaxel	Brain	Normal (3/3)
	Duodenum	Normal (3/3)
	Heart	Normal (3/3)
	Kidney	Normal (3/3)
	Liver	Moderate chronic and acute inflammation (3/3)
Ad-Twist + N-803 + OX40 + 4-1BB + PD-L1	Brain	Normal (3/3)
	Duodenum	Normal (3/3)
	Heart	Normal (3/3)
	Kidney	Normal (3/3)
	Liver	Mild to moderate chronic and acute inflammation (3/3)
Hexatherapy	Brain	Normal (3/3)
	Duodenum	Normal (3/3)
	Heart	Normal (3/3)
	Kidney	Normal (3/3)
	Liver	Mild to moderate chronic and acute inflammation (3/3)

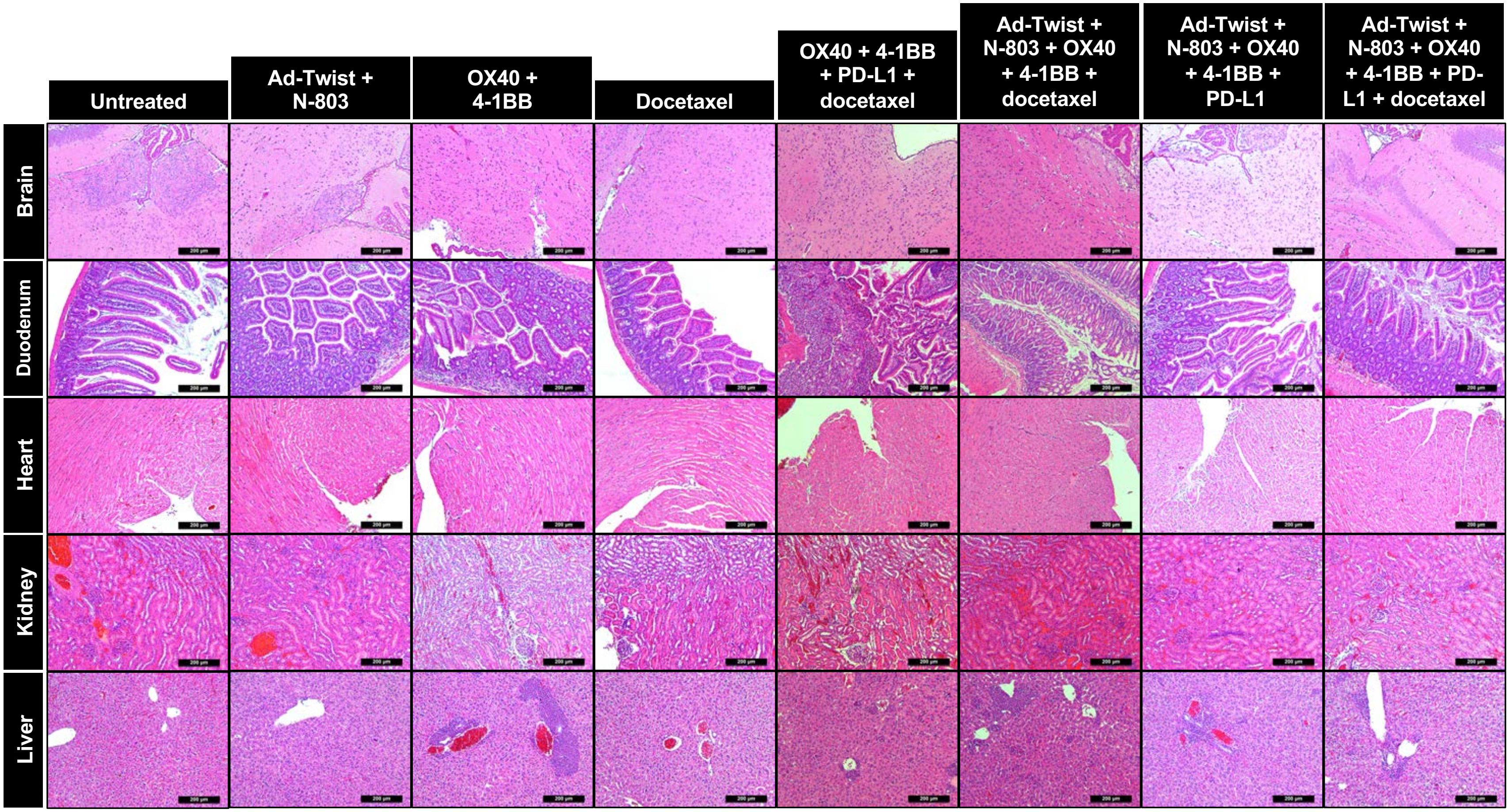

Supplemental Figure 4. Histopathology of organ tissues shows minimal toxicity in the 4T1-bearing mice treated with the hexatherapy regimen. Brain, duodenum, heart, kidney, and liver were collected from 4T1-bearing mice treated with different combination treatments. H&E slides were prepared and examined for abnormalities and signs of combination-related toxicities. PD-L1, programmed death-ligand 1.

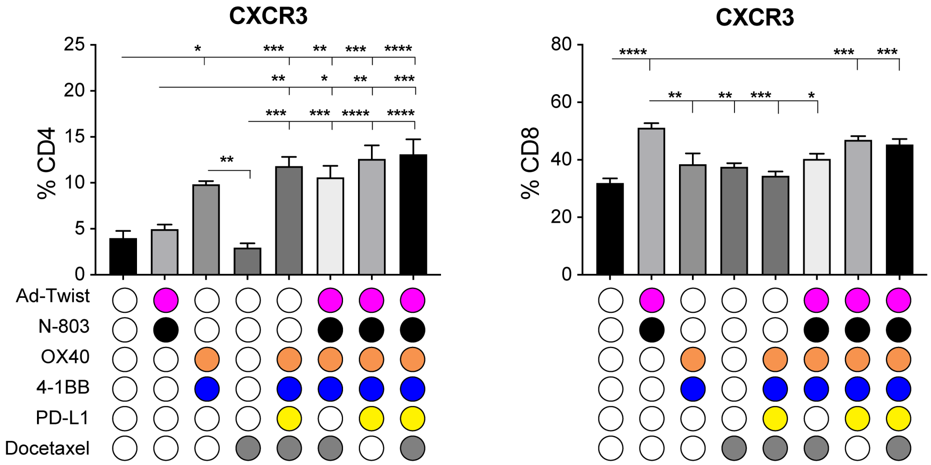

Supplemental Figure 5. Hexatherapy results in increased CXCR3 expression on CD4⁺ and CD8⁺ T cells in the 4T1 tumor model. 4T1-bearing Balb/c mice were treated as described in Figure 4A. Spleens were collected on day 28 post-tumor implantation and flow cytometry was performed to determine the frequency of CXCR3-expressing CD4⁺ and CD8⁺ cells
